# Targeting RIPK1 kinase for modulating inflammation in human diseases

**DOI:** 10.3389/fimmu.2023.1159743

**Published:** 2023-03-08

**Authors:** Wanjin Li, Junying Yuan

**Affiliations:** Interdisciplinary Research Center on Biology and Chemistry, Shanghai Institute of Organic Chemistry, Chinese Academy of Sciences, Shanghai, China

**Keywords:** RIPK1, inflammation, chromatin remodeling, caspase, necroptosis, apoptosis

## Abstract

Receptor-Interacting Serine/Threonine-Protein Kinase 1 (RIPK1) is a master regulator of TNFR1 signaling in controlling cell death and survival. While the scaffold of RIPK1 participates in the canonical NF-κB pathway, the activation of RIPK1 kinase promotes not only necroptosis and apoptosis, but also inflammation by mediating the transcriptional induction of inflammatory cytokines. The nuclear translocation of activated RIPK1 has been shown to interact BAF-complex to promote chromatin remodeling and transcription. This review will highlight the proinflammatory role of RIPK1 kinase with focus on human neurodegenerative diseases. We will discuss the possibility of targeting RIPK1 kinase for the treatment of inflammatory pathology in human diseases.

## Introduction

Inflammation is a common pathological aspect of human diseases, including neurodegenerative diseases. Neuroinflammation has been well-established to be involved in the onset and progression of multiple human chronic neurodegenerative diseases, such as Alzheimer’s disease (AD), Parkinson’s disease, amyotrophic lateral sclerosis and multiple sclerosis (1, 2). The critical issue is how to target neuroinflammation safely and effectively. The nonsteroidal anti-inflammatory drugs (NSAIDs), commonly used in the treatment of peripheral inflammatory diseases such as rheumatoid arthritis, had shown some early promises in delaying the onset of dementia in AD; however, large clinical studies of NSAIDs in AD demonstrated no efficacy and unfavorable side-effects (3–6). In animal and cell models, NSAIDs are known to induce apoptosis (7–9). In this review, we will highlight the role of RIPK1 kinase as a key mediator of sterile inflammation in the CNS and can be safely targeted in mouse models and in humans.

Receptor-Interacting Serine/Threonine-Protein Kinase 1 (RIPK1) has been established as a master regulator of TNFR1 signaling and an important therapeutic target (10). In TNF stimulated cells, RIPK1 controls the cell fate decision whether to promote cell survival or cell death. The activation of RIPK1 kinase activity is involved in mediating pathological cell death and inflammation in a wide arrange of major human diseases from peripheral rheumatoid arthritis and Crohn’s disease to neurodegenerative disorders such as amyotrophic lateral sclerosis and Alzheimer’s disease (10–13). Multiple lines of RIPK1 kinase-dead knockin mutant mice, including D138N, K45A, K584R, S166A and S25D, have been shown to be normal and highly resistant to TNF, while TNF induces cell death and animal lethality in WT mice (14–18). Structurally RIPK1 kinase has a hydrophobic pocket associated with its activation segment that is highly amenable for developing small molecule inhibitors which can stabilize its inactive conformation and allows BBB passage (19–22). Human clinical trials of small molecule RIPK1 inhibitors have demonstrated safety of targeting RIPK1 kinase (23–25). These studies demonstrated the feasibility and safety of targeting RIPK1 in humans.

## RIPK1 is a key mediator of TNFR1 signaling

**Figure 1:**
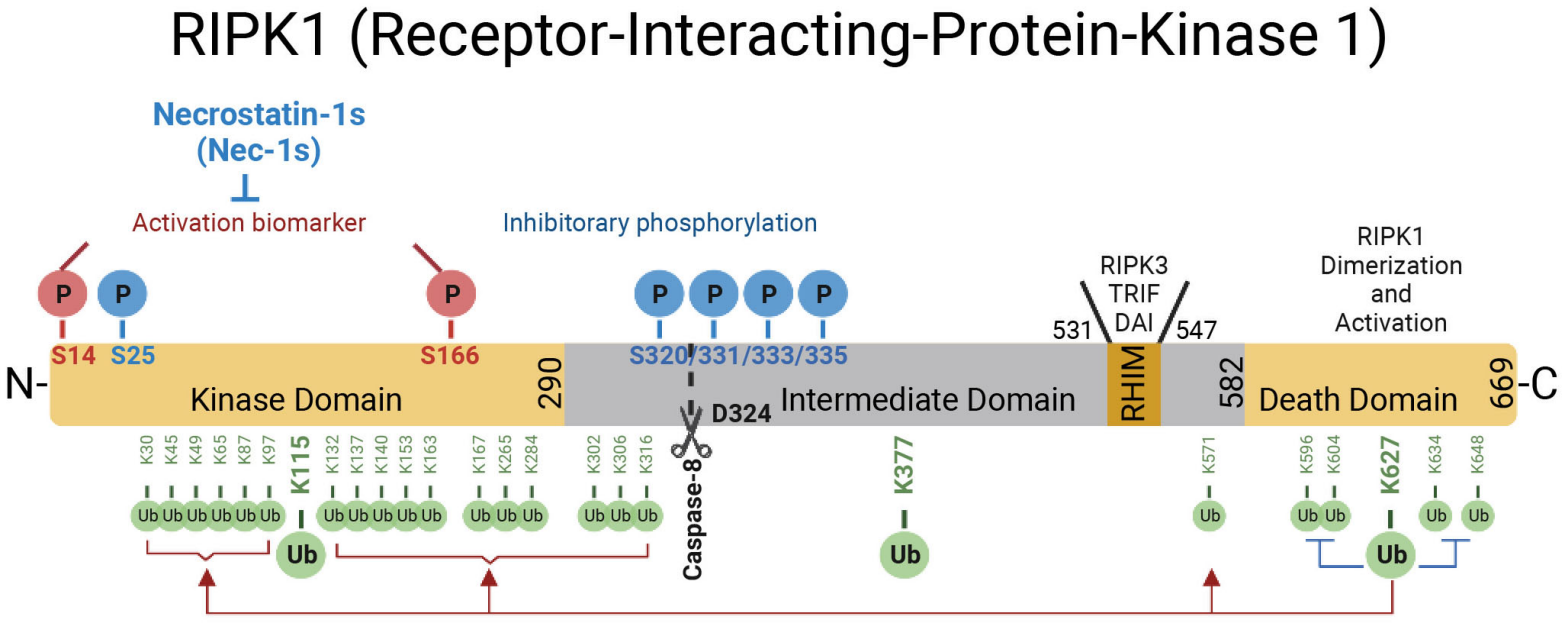
The domains of RIPK1. RIPK1 is a 76-kDa protein with an N-terminal kinase domain, an intermediate domain with a RIP homotypic interaction motif (RHIM), and a C-terminal death domain (DD). The RHIM is required for binding with RIPK3 to mediate necroptosis and other RHIM-containing proteins. The DD is not only crucial for mediating RIPK1 heterodimerization with other DD-containing proteins but also required for RIPK1 activation by mediating RIPK1 dimerization during the transition from complex I to complex II. The top of the RIPK1 kinase depicts the phosphorylation sites in RIPK1. Autophosphorylation of Ser166 is a biomarker for RIPK1 activation. The small molecule Nec-1s is caged in a hydrophobic pocket between the N and C lobes of the kinase domain and forms an H bond between its nitrogen atom and the hydroxyl oxygen of Ser161 on the activation loop to inhibit the activation of RIPK1. The cleavage of RIPK1 after Asp324 by caspase-8 or the phosphorylation on Ser320/331/333/335 in human RIPK1 and Ser321/332/334/336 in murine RIPK1 by TAK1 or MK2 leads to the suppression of RIPK1 activation. As depicted below the RIPK1 kinase, RIPK1 is extensively modulated by ubiquitination with upto 34 ubiquitination sites, especially in the kinase domain and death domain.

**Figure 2:**
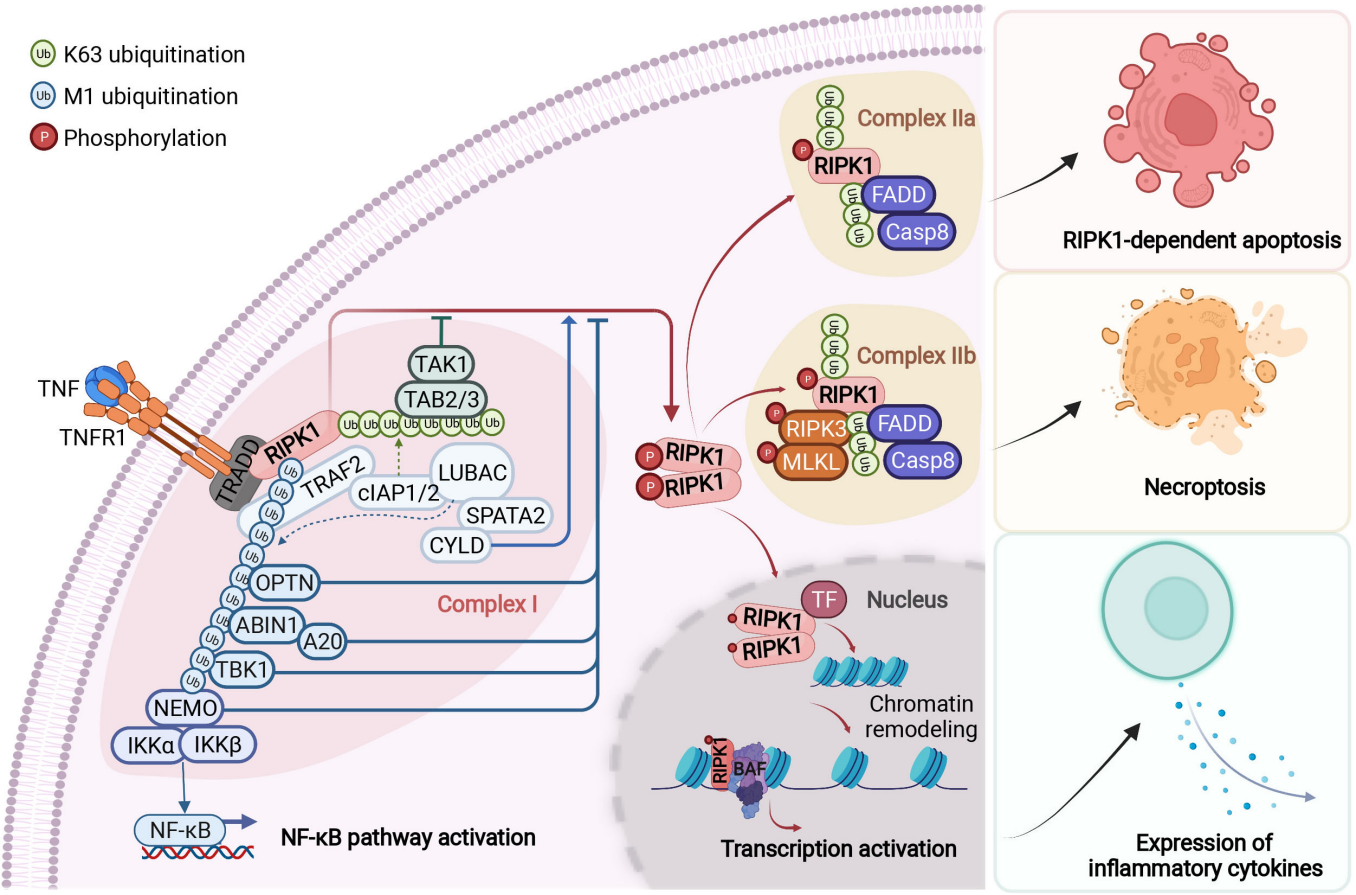
RIPK1-mediated signaling events downstream of TNFR1. Upon binding to tumour necrosis factor (TNF), the cytoplasmic death domain of trimerized TNF receptor 1 (TNFR1) recruits a membrane-associated complex, named complex I. This complex comprises the adaptor protein TNFR1-associated death domain protein (TRADD), the death domain-containing protein kinase receptor-interacting protein 1 (RIPK1) and several ubiquitin E3 ligases, including TNFR-associated factor 2 (TRAF2), cellular inhibitor of apoptosis protein 1 (cIAP1), cIAP2 and the linear ubiquitin chain assembly complex (LUBAC). In complex I, RIPK1 is rapidly polyubiquitylated by Lys63-linked and linear Met1-linked ubiquitin chains, which mediate the recruitment and activation of the TGFβ-activated kinase 1 (TAK1) and IκB kinase (IKK) complexes. The phosphorylation and subsequent ubiquitin–proteasome system (UPS)-mediated degradation of inhibitor of κB (IκB) leads to the activation of nuclear factor- κB (NF-κB). Subsequently, the activity of the deubiquitylation enzymes A20 and cylindromatosis (CYLD) disassembles complex I by deubiquitylating its components, including RIPK1 and TRAF2, within 10–15 minutes after TNF stimulation. This leads to the formation of one of the two alternative cytosolic complexes, complex IIa or complex IIb. Complex IIa includes the adaptor FAS-associated death domain protein (FADD), caspase-8 and RIPK1. The formation of complex IIa mediates the activation of caspase-8. This, in turn, activates downstream caspases, such as caspase-3 (not shown), ultimately leading to apoptosis. When the activation of caspase-8 is inhibited, RIPK1 kinase is activated and binds to RIPK3 to form complex IIb, which mediates the downstream events of necroptosis. The transition from complex I to complex II is an important regulatory step. After disassembled from complex I, RIPK1 is able to translocate to the nucleus and form a RIPK1/BAF complex by directly interacting with two components of BAF, SMARCC2 and BRG1. RIPK1/BAF complex is recruited to the target genomic regions by specific transcription factors upon TNF stimulation, which mediates the chromatin remodeling and transcription activation of proinflammatory genes. NEMO, NF-κB essential modulator; TAB, TAK1-binding protein; TF, transcription factor. Figure is created with BioRender.com.

RIPK1 contains a N-terminal kinase domain, a C-terminal death domain (DD) and an intermediate domain that includes RHIM domain (**Figure 1**). RIPK1 is recruited specifically to the TNFR1 upon its activation by TNF (26, 27) (**Figure 2**). The interaction of RIPK1 with TNFR1 is mediated by homotypic interaction of its DD with the DD in the C-terminus of TNFR1. Another DD-containing adaptor molecule, TRADD, is also recruited to TNFR1 to form TNF-RSC (complex I). TRADD in turn recruits TRAF2 and the E3 ubiquitin ligases cIAP1/2 into complex I to modulate RIPK1 by K63 ubiquitination. K63 ubiquitination of RIPK1 recruits the LUBAC complex containing HOIP, HOIL1, and SHARPIN to perform M1 ubiquitination of RIPK1 which plays an important role in simultaneously mediating the activation of NF-κB pathway by recruiting the ubiquitin binding proteins, such as NEMO, ABIN1, TBK1 and OPTN, as well as IKK complex, and suppressing the activation of RIPK1 (12, 28–30). The polyubiquitin chains in complex I also recruits the ubiquitin binding adaptors TAB2/TAB3 which in turn recruit and activate TAK1, a key kinase involved in the activation of NF-κB pathway (31). These different components of complex I perform extensive ubiquitinating modifications of RIPK1, on as many as 34 lysine residues (32) (**Figure 1**), which collectively decide if the cell should survive by activating NF-κB, or die. The scaffold of RIPK1 has an important function in supporting cell survival by promoting the NF-κB activation, while the activation of RIPK1 kinase activity, regulated by its modifiers in complex I, is pro-death and proinflammation (26, 32–37). The ubiquitination of RIPK1 in complex I serves as the checkpoint I within minutes of TNF stimulation to control the activation of RIPK1 kinase, which may promote proinflammatory cytokine production or cell death including RIPK1 dependent apoptosis (RDA) and necroptosis (**Figure 2**). Dysregulated ubiquitination of RIPK1 in complex I results in the activation of its kinase activity to mediate apoptosis or necroptosis, depending the presence of downstream signaling components such as caspase-8 and RIPK3/MLKL by forming the alternative complex IIa or complex IIb (30, 38–40). While necroptosis as a form of regulated necrosis has been recognized to trigger inflammation (12, 41–44), the activation of RIPK1 kinase activity has been found to promote the transcriptional induction of proinflammatory cytokines but the mechanism was unclear (45–52)(**Figure 2**). A recent report showed that RIPK1 might regulate inflammation by modulating chromatin remodeling directly (53).

The activation of NF-κB pathway suppresses the activation of RIPK1 and cell death mediated by at least two of its transcriptional targets, *TNFAIP3* and *CFLAR* (CASP8 and FADD Like Apoptosis Regulator, also called *c-FLIP*) (54, 55). In cells stimulated by TNF, *TNFAIP3*, transcriptionally induced by the NF-κB pathway, encodes the ubiquitin editing enzyme A20, which acts an upstream regulator of RIPK1 by modulating its ubiquitination pattern (56). Thus, A20 serves as a key ubiquitinating modifying enzyme in the checkpoint I that controls the activation of RIPK1 (**Figure 2**). On the other hand, *CFLAR* encodes multiple c-FLIP isoforms which share similarity in amino acid sequence with caspase-8 but lack the caspase activity, and at least some of the c-FLIP isoforms can directly bind to caspase-8 to inhibit its activation (57). Since the cleavage of human RIPK1 at D324 and murine RIPK1 at D325 controls the activation of the kinase by separating it from the C-terminal DD which can dimerize to promote the N-terminal kinase activation (16, 58) (**Figure 1**), caspase-8 is a downstream negative regulator of RIPK1 kinase. Thus, the cleavage of RIPK1 mediated by caspase-8 may serve as the checkpoint II in TNF stimulated cells that controls the activation of RIPK1 (**Figure 2**). Expression of RIPK1 mutants D325V or D325H, which block the cleavage of RIPK1 by caspase-8, sensitize cells to RIPK1 activation-mediated apoptosis and necroptosis, as well as induce the production of pro-inflammatory cytokines such as IL-6 and TNF (52, 59). Thus, the activation of RIPK1 and NF-κB may cooperate to mediate inflammatory response.

## RIPK1 regulates inflammatory response in human diseases

While the role of RIPK1 in regulating cell death has been well-established, the recent studies highlighted its role in mediating proinflammatory responses. Heterozygous mutations that altered Asp324 of RIPK1 in human lead to early-onset periodic fever syndrome, defined as cleavage-resistant RIPK1-induced autoinflammatory syndrome (CRIA) (46, 48, 52). In particular, the expression of RIPK1 noncleavable variants, such as D324V and D324H, in humans, which blocks the cleavage by caspase-8, leads to an autoinflammation clinically characterized by recurrent fevers and lymphadenopathy (46, 52). Overproduction of inflammatory cytokines and chemokines were detected in the blood of these patients and the recurrent fevers in the patients respond partially to the treatment of anti-IL6. The expression of the caspase-8 cleavage-resistant RIPK1 (*Ripk1^D325A/D325A^*) resulted in embryonic lethality around E10.5 (46, 48). Though *Mlkl^−/−^Fadd^−/−^ or Ripk3^−/−^caspase-8^−/−^* mice are able to fully rescue *Ripk1^D325A/D325A^* animals, there were characteristic autoimmune lymphoproliferative syndrome (ALPS) observed in the surviving mice, suggesting the role of activated RIPK1 in mediating inflammation independent of necroptosis (46, 48, 49, 60, 61). In animal models and human pathological studies, the activation of RIPK1 has also been implicated in mediating inflammation in major human chronic inflammatory diseases including rheumatoid arthritis, inflammatory bowel disease and psoriasis (10, 62–65).

M1 ubiquitination of RIPK1 mediated by LUBAC complex plays a critical role in restraining RIPK1 kinase activity in skin inflammation. LUBAC is a key regulator of TNFR1 mediated cell death and inflammation (29, 66–69). Mice deficient for the gene encoding the LUBAC regulatory subunit SHARPIN, known as the chronic proliferative dermatitis mice (*cpdm)* (*Sharpin^cpdm/cpdm^* mice), suffer from severe multi-organ inflammation particularly in the skin that resembles atopic dermatitis and psoriasis in humans (15, 70). Inhibition of RIPK1 by GNE684 or RIPK1^D138N/D138N^ protects against skin inflammation in *cpdm* mice (15, 44, 71). OTULIN is a deubiquitinating enzyme that plays a critical role in regulating LUBAC activity (72). Biallelic hypomorphic mutations in *OTULIN* in humans lead to a severe form of autoinflammatory disease, known as OTULIPENIA or ORAS (73, 74). Mice with epidermis-specific loss of OTULIN develop inflammatory skin lesions that can be inhibited by TNFR1 deficiency and inhibition of RIPK1 kinase (75).

The activation of RIPK1 has also been implicated in mediating inflammatory responses in neurodegenerative diseases such as amyotrophic lateral sclerosis (ALS), multiple sclerosis (MS) and Alzheimer’s disease. Pathological evidence for neuroinflammation in ALS, a progressive neurodegenerative disease that affects motor neurons in the brain and the spinal cord, include changes in circulating immune cell populations and cytokines, which can be noted in the early stage of the disease (76). Loss of *OPTN*, a monogenic cause of ALS, leads to RIPK1-dependent microglial activation, dysmyelination and necroptosis of motor neurons in the spinal cords of OPTN^-/-^ mice, which was rescued by genetic and pharmacological inactivation of RIPK1 kinase (77). OPTN deficient microglia present an activated inflammatory state which can be suppressed upon inhibition of RIPK1. A single-cell RNA-seq study identified a class of microglia, termed RIPK1-Regulated Inflammatory Microglia (RRIMs), in the early stage of ALS that show significant up-regulation of classical proinflammatory pathways in RIPK1-dependent manner (78).

MS is an autoimmune disease of the CNS that involves RIPK1 activation (79). TNFα is strongly linked with the etiology of MS (80, 81). The loss of myelinating oligodendrocytes, which normally insulate the axons of neurons, is a hallmark of MS. Necroptosis is the primary mode of cell death for oligodendrocytes in the presence of TNFα stimulation alone (79). TNFAIP3 has been identified as a risk gene in a large genetic association study highlighting the contribution of peripheral immune cells and microglia to MS (82). Down-regulation of caspase-8 levels has been noted in the cortical pathological samples from MS patients (79). Thus, the pathology of MS may include the defects in both checkpoints I and II that control the activation of RIPK1. Genetic (RIPK1^D138N/D138N^) and pharmacological (Nec-1s) inhibition of RIPK1 kinase ameliorated disease pathology, improved animal behavior, attenuated the production of proinflammatory cytokines, and decreased recruitment of immune cells in multiple animal models of MS (79, 83).

RIPK1 may also play an important role in driving neuroinflammation in AD (84). In microglia of mouse models of AD, the activation of RIPK1 drives transcriptional induction of *Cst7*, which encodes an endosomal/lysosomal cathepsin inhibitor named Cystatin F, a biomarker for disease-associated microglia (DAM) present in spatial proximity to Aβ plaques in both postmortem human AD brain samples and in an AD mouse model (85). Inhibition of RIPK1 reduces neuroinflammation and cognitive deficits in the APP/PS1 amyloid β-driven mouse model of AD (84).

RIPK1-mediated inflammatory response has also been shown to be involved in severe cases of Coronavirus disease 2019 (COVID-19) caused by severe acute respiratory syndrome coronavirus 2 (SARS-CoV-2) (86). SARS-CoV-2 poses substantial challenges to public health worldwide mainly due to its ability to promote life-threatening systemic inflammatory response in a subset of COVID-19 patients. Activated RIPK1 was found in human COVID-19 lung pathological samples, respiratory tract epithelial cells from COVID-19 patients, cultured human lung organoids and ACE2 transgenic mice infected by SARS-CoV-2 (86, 87). Inhibition of RIPK1 using multiple small-molecule inhibitors reduced the viral load in human lung organoids infected by SARS-CoV-2. The SARS-CoV2 3C-like protease (3CLpro) can cleave NEMO and promote RIPK1-mediated endothelial cell death and BBB-damage in COVID-19 (88, 89). The viral load in lung and mortality and is reduced by the treatment of RIPK1 therapeutic inhibitor Nec-1s, blocking SARS-CoV-2 manifestation in the CNS of ACE2 transgenic mice (86). NSP12 is an RNA-dependent RNA polymerase of SARS-CoV-2, which is important in mediating coronaviral transcription and replication. The C14408T variant of SARS-CoV-2, first reported in Italy and present in all subsequently evolved strains including Delta and Omicron, encodes NSP12 323L variant. This 323L variant of NSP12 induced higher activation level of RIPK1 compared to wildtype NSP12. Notably, the transcriptional expression level of host factors promoting viral entry such as EGFR and ACE2 as well as proinflammatory genes can be downregulated with inhibition of RIPK1. Thus, SARS-CoV-2 may be able to hijack the RIPK1-mediated host defense response to its advantage and that inhibition of RIPK1 may provide a therapeutic option for the treatment of severe COVID-19 to help ending this public health emergency.

## Activated RIPK1 mediates sustained inflammatory response involving NF-κB

TNFα-induced necroptosis leads to two waves of cell-autonomous proinflammatory cytokine production (51). The first wave, transient and weak in nature, is activated in response to TNFα alone; whereas the second wave, stronger and sustained, depends upon the necroptotic signaling and the activation of RIPK1. In contrast, the level of cytokine production promoted by a direct oligomerization of MLKL-induced necroptosis is much lower than that by TNFα-induced necroptosis. Thus, the activation of RIPK1 can also mediate inflammatory response during necroptosis.

The activation of inflammation by lipopolysaccharide (LPS) may also involve such RIPK1-mediated biphasic production of inflammatory response (90). Upon inhibition of caspases in cells stimulated by LPS, a biphasic production of proinflammatory cytokines has been noted. With caspase inhibition, the early production of LPS stimulated proinflammation cytokines is independent of RIPK1 kinase activity but dependent upon the scaffold function of RIPK1 involving in the NF-κB pathway. Another TNFR1-associated complex containing key mediators of NF-κB, FADD, activated RIPK1 and caspase-8 is formed upon the stimulation of autocrine TNFα produced from the early phase. In contrast with the early phase, the second phase of proinflammatory cytokine production is dependent of RIPK1 kinase activity as it can be blocked with pharmacological inhibition of RIPK1 kinase. Interestingly, such biphasic mode of proinflammatory cytokine production has also been previously reported in cells stimulated by TRAIL, another member of TNF family (91). Therefore, the scaffold function of RIPK1 is promoted by its kinase activation to regulate the proinflammatory cytokine induction mediated by NF-κB signaling with inhibition of caspases. Since the cleavage of RIPK1 after the kinase domain at D324 hRIPK1 and D325 mRIPK1 by caspase-8 negatively regulates its kinase activation, the periodic fever episodes of rare human carriers of non-cleavable RIPK1 may involve their interaction with TLR ligands which may cause minimal inflammation normally inconsequential in normal individuals (46, 52).

cIAP1/2 mediated-ubiquitination of RIPK1 recruits adaptors TAB1/2 to support TAK1 (Transforming growth factor-α-activated kinase 1, also called MAP3K7) activation (31). Activated TAK1 then mediates the phosphorylation IKK complex consisting of IKKα/β/γ (NEMO) to support its formation (92). Interestingly, RIPK1 is a phosphorylation substrate of TAK1 as well as IKKs (93, 94). IKKs mediated S25 phosphorylation on RIPK1 negatively regulates the activation of RIPK1 kinase activity and RIPK1-dependent cell death. TAK1 mediates the phosphorylation of multiple sites in the intermediate domain of RIPK1 including S321 (93). Phospho-Ser321 (S321) RIPK1 is a biomarker for phosphorylation of RIPK1 by TAK1. While the S321A mutation per se has no effect on the NF-κB activation, the loss of TAK1-mediated phosphorylation of RIPK1 promotes its binding to FADD and RIPK1-dependent apoptosis (RDA); furthermore, sustained TAK1-mediated phosphorylation of RIPK1 promotes its interaction with RIPK3 to mediate necroptosis (93, 95). Thus, there are intriguing interactions of upstream activators of NF-κB, TAK1 and IKKs, with RIPK1, whose scaffold also functions in mediating the activation of NF-κB pathway. The intertwined relationship between RIPK1 and NF-κB pathway may promote inflammatory responses in a temporal specific manner: the activation of NF-κB pathway initiates an early inflammatory response while the activation of RIPK1 is important for mediating sustained chronic inflammation.

TBK1 (TANK binding kinase 1) is known as a key regulator of type I IFN, NF-κB and TNF-mediated RIPK1-dependent cell death (96). Rare human individuals with homozygous loss-of-function mutations in TBK1 suffer from chronic systemic autoinflammation (97). Interestingly, the loss of TBK1 in humans leads to reduced IFN-I production but nevertheless adequate antiviral response, and nearly normal NF-κB-mediated IL6 production, suggesting that the role of TBK1 in regulating IFN-I and NF-κB signaling may be insufficient to explain the autoinflammatory phenotype. Biallelic loss of TBK1 in mice is embryonic lethal which can be effectively suppressed by knockout of TNF or TNFR1, and by genetic inactivation of RIPK1 (30, 98). TBK1 can directly inhibit the activation of RIPK1 kinase by preforming inhibitory phosphorylation on RIPK1 (29, 30). The loss of TBK1 sensitized to RIPK1-dependent cell death in both mouse and human cells which may be responsible for the autoinflammatory pathology in TBK1 deficient individuals (30, 97). In addition, another recent study reported the role of RIPK1/RIPK3-mediated inflammatory cascade of alloreactive T cell responses in promoting graft-versus-host disease (GVHD) (99). Inhibition of RIPK1 was able to reduce the expression of proinflammatory cytokines and MHC class II molecules in intestinal epithelial cells and arrest GVHD without compromising the graft-versus-leukemia (GVL) effect. Therefore, targeting RIPK1/RIPK3 in IECs provides a strategy for GVHD treatment without compromising the normal reaction of immune system. These studies suggest the contribution of necroptosis to inflammatory responses in different human pathological conditions.

Heterozygousity in TBK1 has been linked with the onset of familial amyotrophic lateral sclerosis (ALS) and frontotemporal dementia (FTD) in aging (100–102). Genetic interaction of specific mutations with aging is associated with a variety of neurodegenerative diseases. Since both TBK1 and TAK1 preform inhibitory phosphorylation on RIPK1, the interaction of TAK1 and TBK1 in aging was explored (30, 93). The reduction of TAK1 expression in aging human brains was shown to cooperate with heterozygous loss-of-TBK1 to promote late onset ALS/FTD-like pathology mediated by decreased RIPK1 inhibition. Reduced expression of TAK1 in aging human brains may promote RIPK1-mediated inflammatory response to cooperate with additional pre-existing conditions to mediate the onset of neurodegeneration. Thus, inhibiting RIPK1 kinase may provide an effective strategy for reducing chronic inflammation that underlies a variety of aging-related human diseases.

## Chromatin remodeling in inflammatory responses

Activation of inflammatory responses involves very elaborated mechanisms to ensure a coordinated program that regulates the expression of induced genes in a temporally and spatially controlled manner. Regulation of inflammatory responses by NF-κB, which has been extensively studied, provides such an example. The interaction of master regulators of NF-κB pathway with chromatin is a key step in driving the complex and diverse gene expression programs (103). Mammalian genomic DNA is tightly packed into chromatins (104, 105). Chromatin remodeling complexes were found to be important for granting dynamic access to the highly packaged DNA and to remodel the nucleosome composition on their target regions. Chromatin remodeling allows accessibility and transcription of specific genes, by precisely controlling the dynamics of chromatin (106–111). Thus, it is not surprising that chromatin remodeling is involved in the transcriptional induction of proinflammatory factors.

BRG1/BRM-associated factor (BAF) chromatin-remodeling complex, the mammalian homolog of the SWI/SNF complex in yeast, is an important regulator of gene expression. It regulates higher-order chromatin organization that modulate chromatin accessibility by displacing the positions of nucleosomes in an ATP-dependent manner in response to specific developmental and environmental signals (111, 112). While SWI/SNF complex has been demonstrated to be involved in tumorigenesis (113, 114) and in neural development and disorders (105), recent studies have showed the role of SWI/SNF complex in the regulation of inflammatory responses. SWI/SNF complex is shown to be recruited to the promoters of IFN-γ induced genes (115–117) and is critically important in the transcriptional induction of LPS-induced inflammatory genes (118–122). LPS induces histone modifications and chromatin remodeling to allow NF-κB to gain access to the IL-12 promoter and other NF-κB-dependent promoters (123, 124). BRG1, the catalytic subunit of SWI/SNF, is recruited by MALAT1 to the promoter region of *IL-6* and *CXCL8* and interacts with NF-κB to promote LPS-induced inflammatory factors production in HCC cells (125). BRG1 is also involved in LPS or endotoxin tolerance. In LPS tolerant macrophages, TLR inducible recruitment of BRG1 and chromatin remodeling are lost on the promoters of proinflammatory genes which are not responsive to re-stimulation with LPS (126). BRG1 plays an important role in T cell development, differentiation and transcription of cytokines (127–133). The enrichment of BRG1 on chromatin dynamically changes during T cell activation and varies in different types of T cells. BRG1 promotes inflammation in CD4 cells while the Treg-specific deletion of BRG1 impairs Treg activation and results in early-onset fatal inflammation (134). The recruitment of SWI/SNF complex to the promoters or enhancers of proinflammatory genes can be mediated by transcriptional activators (135, 136), long noncoding RNAs (lncRNAs) (137–139), histone modifications (140, 141) or the mediator complex (122, 141–143). Thus, the binding specificity of SWI/SNF complex in different tissue or cells upon various stimulation is directed by the factors recruiting them.

## Nuclear RIPK1 promotes inflammatory response by mediating chromatin remodeling

The extensive involvement of RIPK1 in mediating inflammatory responses raises the question how activation of RIPK1 may promote the expression of pro-inflammatory cytokines. Using p-S166-RIPK1 as a biomarker of RIPK1 activation (21, 79), activated RIPK1 is detected in both nucleus and cytoplasm of MEFs upon stimuli causing RDA or necroptosis which is associated with the induction of pro-inflammatory cytokines (51, 52). Nuclear RIPK1 activation is also observed in spinal cords of ALS patients (53). RIPK1 has also been detected in nucleus during TNF-induced necroptosis (144, 145) and H_2_O_2_-induced oxidative cell death (146). Interestingly, blocking necroptosis by RIPK3 knockout or MLKL knockout could not inhibit the nuclear translocation of activated RIPK1 or RIPK1-mediated induction of inflammatory gene expression, suggesting that RIPK1 mediated cell death can be mechanistically separated from that of RIPK1-regulated inflammation (53).

The genomic targets of activated RIPK1 in RIPK1 D325A MEFs, as well as in spinal cords from human ALS patients and *Tbk1^+/-^; Tak1^ΔM/+^* ALS mouse model (30, 53), are highly enriched in inflammatory responses, suggesting that nuclear RIPK1 directly participates in the regulation of specific gene transcription. The nuclear interactome of RIPK1 includes a striking collection of transcriptional activators/coactivators, suggesting that nuclear RIPK1 may be directly involved in mediating transcriptional activation. Interestingly, the nuclear interacting partners of RIPK1 includes p65 (RELA), the key transcription factor in the canonical NF-κB pathway and other transcription factors that have been found to promote inflammation, such as SP1, JUNB (147–151), suggesting that the activated RIPK1 interacts with multiple transcriptional factors in nucleus to mediate inflammation.

Targeting BAF complex to specific genomic regions during development is known to involve the interactions with transcriptional factors including the members of NF-κB family and JUN (152). Activated nuclear RIPK1 was found to interact with almost all components of BAF complex, including BRG1 and SMARCC2. Thus, the binding of activated RIPK1 with the transcriptional factors and BAF complex integrates the external TNF signaling to the specific genomic regions on chromatin in nucleus to modulate chromatin dynamics for activating an inflammatory response.

**Figure 3:**
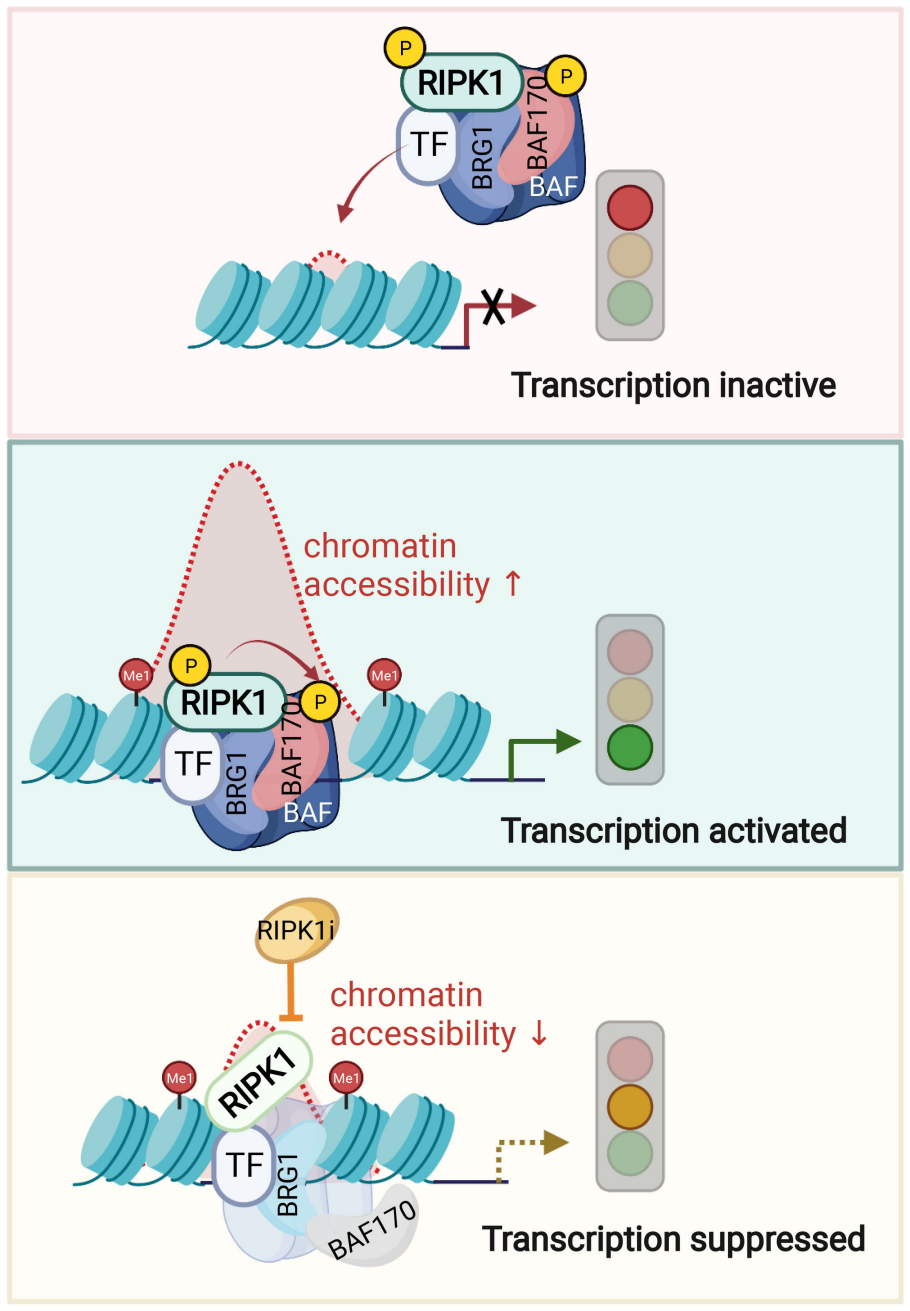
Nuclear RIPK1 promotes transcription of inflammatory cytokines by modulating chromatin remodeling activity. A model of RIPK1 kinase activity dependent transcriptional activation by regulating chromatin modeling activity. Upon TNFα stimulation, transcription factors recruit RIPK1/BAF complex to the genomic target sites. RIPK1 interacts with BRG1 and SMARCC2 directly. Activated RIPK1 mediates the phosphorylation of SMARCC2, which is important in promoting the BAF complex-medicated chromatin remodeling. Consequently, the targeting enhancer DNA accessibility is increased, and the transcription of target genes is activated. Inhibition of RIPK1 suppresses the phosphorylation of SMARCC2, which disrupts the co-occupancy of SMARCC2 and BRG1 on the target sites and as a result, suppresses the transcription of proinflammatory factors by reducing the enhancer DNA accessibility. Figures are created with BioRender.com.

Phosphorylation of BAF complex is known to modulate its activity in response to specific signaling. ATM kinase, when activated by DNA damage, can phosphorylate BRG1 to stimulate BRG1 nucleosome binding activity by enhancing its affinity for H3K14ac (153). In TNF stimulated condition, nuclear RIPK1 directly phosphorylates Ser306 of SMARCC2, a key scaffolding subunit of BAF complex. p-S166 RIPK1, BRG1 and SMARCC2 co-occupation was found on the key TNF induced accessible genomic regions of proinflammatory genes (53). The DNA accessibility and co-occupancy of BRG1 and SMARCC2 upon TNF in RIPK1 D325A mutant MEFs at p-S166 RIPK1 binding sites are both reduced upon inhibition of RIPK1 kinase. Thus, activated RIPK1 in the nucleus of TNF stimulated cells is recruited by transcription factors to interact with BAF and form a RIPK1/BAF complex to regulate chromatin remodeling activity and the accessibility to specific genomic DNA regions to promote the transcription of proinflammatory genes (**Figure 3**).

## Conclusions and remaining questions

The past two decades of research have provided ever expanding knowledge on RIPK1 biology, which led to the appreciation on the importance of RIPK1 activation in the cell types that are key mediators of proinflammatory response in a variety of pathological conditions, such as macrophages and microglia. Activation of RIPK1 has been shown to mediate not only apoptosis and necroptosis in TNF-stimulated condition but also the activation of proinflammatory responses (51, 90). While necroptosis has been shown to be proinflammatory, the functional consequence of RIPK1 activation in mediating transcriptional induction of inflammatory response independent of cell death may be mechanistically separated from circumstances where cell death is present. Recent studies have begun to demonstrate the mechanism by which nuclear RIPK1 directly participates in the transcriptions upon recruitment by specific transcriptional factors and chromatin remodeling BAF complexes independent of cell death (53). We suggest that since inflammation represents a reversible cellular state, unlike that of cell death which can lead to permanent loss or reduction of a certain cell population, targeting RIPK1 kinase for inhibiting its nuclear function in modulating BAF-mediated chromatin remodeling may represent a clinically important paradigm where inflammation is the dominant pathology. Human clinical studies using small molecule inhibitors of RIPK1 are currently underway for the treatment of human conditions that involve cell death and inflammation, e.g. ALS, AD and MS etc. (10). Importantly, the nuclear function of RIPK1 in driving inflammatory response suggests that small molecule inhibitors of RIPK1 should be considered for the treatment of human conditions that are inflammatory in nature and may not include cell death. However, many questions remain to be answered by future studies. For example, what might be the mechanism that regulates the translocation of RIPK1 from cytoplasm to nucleus? If and how the nuclear translocation of RIPK1 is coupled with its activation? What might be the substrates of RIPK1 in the nucleus? Can we manipulate the subcellular location of activated RIPK1 in order to modulate the process of RIPK1-dependent cell death and inflammation precisely? The answers to these questions will help us to further understand the mechanism by which RIPK1 regulates cell death and inflammation and better develop the therapeutic strategies for the treatment of human inflammatory and neurodegenerative diseases.

## Author contributions

This review was written by JY and WL. All authors contributed to the article and approved the submitted version.
